# Complementary Metagenomic Approaches Improve Reconstruction of Microbial Diversity in a Forest Soil

**DOI:** 10.1128/mSystems.00768-19

**Published:** 2020-03-10

**Authors:** L. V. Alteio, F. Schulz, R. Seshadri, N. Varghese, W. Rodriguez-Reillo, E. Ryan, D. Goudeau, S. A. Eichorst, R. R. Malmstrom, R. M. Bowers, L. A. Katz, J. L. Blanchard, T. Woyke

**Affiliations:** a Graduate Program in Organismic and Evolutionary Biology, University of Massachusetts Amherst, Amherst, Massachusetts, USA; b Department of Energy, Joint Genome Institute, Berkeley, California, USA; c Centre for Microbiology and Environmental Systems Science, Department of Microbiology and Ecosystem Science, Division of Microbial Ecology, University of Vienna, Vienna, Austria; d Department of Biological Sciences, Smith College, Northampton, Massachusetts, USA; e Department of Biology, University of Massachusetts Amherst, Amherst, Massachusetts, USA; Pacific Northwest National Laboratory

**Keywords:** flow cytometry, metagenomics, microbial ecology, soil microbiology

## Abstract

Soil ecosystems harbor diverse microorganisms and yet remain only partially characterized as neither single-cell sequencing nor whole-community sequencing offers a complete picture of these complex communities. Thus, the genetic and metabolic potential of this “uncultivated majority” remains underexplored. To address these challenges, we applied a pooled-cell-sorting-based mini-metagenomics approach and compared the results to bulk metagenomics. Informatic binning of these data produced 200 mini-metagenome assembled genomes (sorted-MAGs) and 29 bulk metagenome assembled genomes (MAGs). The sorted and bulk MAGs increased the known phylogenetic diversity of soil taxa by 7.2% with respect to the Joint Genome Institute IMG/M database and showed clade-specific sequence recruitment patterns across diverse terrestrial soil metagenomes. Additionally, sorted-MAGs expanded the rare biosphere not captured through MAGs from bulk sequences, exemplified through phylogenetic and functional analyses of members of the phylum *Bacteroidetes*. Analysis of 67 *Bacteroidetes* sorted-MAGs showed conserved patterns of carbon metabolism across four clades. These results indicate that mini-metagenomics enables genome-resolved investigation of predicted metabolism and demonstrates the utility of combining metagenomics methods to tap into the diversity of heterogeneous microbial assemblages.

**IMPORTANCE** Microbial ecologists have historically used cultivation-based approaches as well as amplicon sequencing and shotgun metagenomics to characterize microbial diversity in soil. However, challenges persist in the study of microbial diversity, including the recalcitrance of the majority of microorganisms to laboratory cultivation and limited sequence assembly from highly complex samples. The uncultivated majority thus remains a reservoir of untapped genetic diversity. To address some of the challenges associated with bulk metagenomics as well as low throughput of single-cell genomics, we applied flow cytometry-enabled mini-metagenomics to capture expanded microbial diversity from forest soil and compare it to soil bulk metagenomics. Our resulting data from this pooled-cell sorting approach combined with bulk metagenomics revealed increased phylogenetic diversity through novel soil taxa and rare biosphere members. In-depth analysis of genomes within the highly represented *Bacteroidetes* phylum provided insights into conserved and clade-specific patterns of carbon metabolism.

## Abstract (precis)

Microbial ecologists have historically used cultivation-based approaches as well as amplicon sequencing and shotgun metagenomics to characterize microbial diversity in soil. However, challenges persist in the study of microbial diversity, including the recalcitrance of the majority of microorganisms to laboratory cultivation and limited sequence assembly from highly complex samples. The uncultivated majority thus remains a reservoir of untapped genetic diversity. To address some of the challenges associated with bulk metagenomics as well as low throughput of single-cell genomics, we applied flow cytometry-enabled mini-metagenomics to capture expanded microbial diversity from forest soil and compare it to soil bulk metagenomics. Our resulting data from this pooled-cell sorting approach combined with bulk metagenomics revealed increased phylogenetic diversity through novel soil taxa and rare biosphere members. In-depth analysis of genomes within the highly represented *Bacteroidetes* phylum provided insights into conserved and clade-specific patterns of carbon metabolism.

## INTRODUCTION

Soil is considered to be among the most biologically diverse ecosystem types, and yet much of its microbial diversity remains poorly characterized (see, e.g., references 1 and 2). Each gram of soil is estimated to harbor 1,000 to 1,000,000 different bacterial species (see, e.g., references 3 to 7). Investigating soil microorganisms *in situ* is challenging due to the heterogeneous nature of the soil environment (see, e.g., references 8 to 10). As a result, terrestrial habitats remain immense reservoirs of untapped genetic and metabolic diversity (7, 11) encoded within microbial communities that drive important ecosystem-level processes, including nitrogen cycling and carbon dioxide flux (12–14). Soils are regarded as critical for global health, as they contain 3,000 Pg of carbon and have the potential to act as either a carbon source or a carbon sink, which is important to consider under conditions of climatic shift (15, 16). It is therefore essential to characterize soil microbial diversity to better understand ecosystem function and resilience in the face of rapid environmental change.

Historically, microbial diversity has been studied using laboratory cultivation techniques (17, 18) with only a minute fraction of estimated bacterial diversity being successfully cultivated. Substantial efforts are being made to develop innovative cultivation techniques, including the ichip and droplet-based sorting coupled with laboratory cultivation (17, 19). These approaches have contributed to expansion of diversity within novel families. However, cultivation-independent investigations may further our understanding of microbial diversity by facilitating description of novel higher taxonomic ranks. Thus, challenges associated with direct study of soil microorganisms have yielded a large knowledge gap regarding terrestrial microbial diversity. Due to limitations associated with cultivation, relatively few isolate genomes are available as references for soil microbes (20). From the publicly available Integrated Microbial Genomics (IMG/M) database (21), we were able to curate a collection of 3,024 isolate genomes, single amplified genomes (SAGs), and metagenome assembled genomes (MAGs) from previous soil studies. However, with soil estimated to contain 1,000 to 1,000,000 species per gram (9), these references represent only a small percentage of soil microbes.

In addition to culture-based approaches, amplicon studies have greatly contributed to our knowledge of microbial community structure (1, 22). However, amplicon sequencing primers that target the small-subunit (SSU) rRNA gene may not adequately amplify some organisms due to primer biases through mismatches (22). Additionally, estimates of organismal abundance may be conflated by variation in gene copy number (23). Phylogenetically divergent taxa may be overlooked using PCR-based approaches, thereby hampering our ability to describe an expanded diversity of organisms (22). High-throughput sequencing technologies combined with novel metagenome binning algorithms (24, 25) enable genome-resolved metagenomics studies and have greatly expanded the availability of reference genomes from uncultured taxa by circumventing challenges associated with cultivation- and amplicon-based studies (11, 26, 27). The more recent applications of directly sequencing DNA from soil microbial communities allow one to obtain a broader perspective on the taxonomic and functional potential of soil microorganisms. However, metagenomics in highly diverse environments may capture only the most abundant and therefore best-assembling representatives from the total community (28–30), and population heterogeneity can hamper the efficiency of assembly, even of abundant microorganisms (31).

Population microheterogeneity of closely related strains within microbial communities makes the separation of individual strains challenging (32). Soils are typically dominated by a small set of highly abundant taxa (12), and the rare biosphere may therefore be overlooked in metagenomic studies despite playing an important role in soil biogeochemical processes (33). Lastly, bulk metagenomics can also include extracellular DNA from dead microorganisms, which may be abundant in the environment. the presence of this exogenous DNA has the potential to inflate estimates of diversity and genomic potential (34–36) and to further reduce our ability to assemble sequences from rare taxa. Decoupling intracellular and exogenous DNA during sequencing may provide a more accurate estimate of microbial diversity (36).

Challenges associated with bulk metagenomics may be mitigated by reducing community complexity. The most extreme example involves the application of fluorescence-activated cell sorting (FACS) for separating communities into single cells for single-cell genomics, which provides genomic information with strain-level resolution (37–39). However, the resulting SAG assemblies are often highly fragmented and incomplete, and the overall process is prone to biases and contamination. In order to circumvent some of the challenges associated with bulk metagenomics and single-cell genomics, we applied a pooled-cell sorting approach coupled to shotgun sequencing, termed mini-metagenomics, to forest soils collected from the Barre Woods soil warming experiment at the Harvard Forest Long-Term Ecological Research (LTER) site. This mini-metagenomic approach separates a researcher-defined number of cells from the larger community, which then undergo lysis and whole-genome multiple-displacement amplification (MDA), followed by sequencing.

Prior to the application of cell sorting to Harvard Forest soil in this study and in that by Schulz et al. (40), mini-metagenomics analysis of microorganisms had been used only in aqueous environments, including hot springs, hospital sink biofilms, and activated sludge (40–44). Mini-metagenomics has higher throughput than single-cell genomics, providing the opportunity to capture more diversity than is possible with single-cell sequencing. Mini-metagenomics may enable investigation of different components of the soil community in comparison to bulk metagenomics, including cells that can be dissociated from particles, and cells with susceptibility to the single-cell lysis step. The use of two overlapping metagenomic methods may allow us to capture a broader taxonomic diversity than the use of only one approach on its own. Additionally, cell sorting using FACS requires cells to be intact in order to be sorted, thereby minimizing challenges introduced by extracellular DNA in bulk soil samples. Using mini-metagenomics to reduce the number of cells relative to bulk metagenomics may decrease the number of genomes collapsed into a single MAG (41). Hence, we evaluated this method as a tool to complement bulk metagenomics in uncovering the “microbial dark matter” in soil.

Here, we combined mini-metagenomics and bulk metagenomics as complementary approaches for capturing a more holistic perspective of microbial community diversity. We discovered additional diversity of uncultivated microorganisms in a forest soil microbial community and thus contribute to the known diversity of both major soil clades and understudied taxonomic groups, which can be used as reference sequences in future studies. Additionally, we provide an example of how the mini-metagenomics and bulk metagenomic approaches can be used in complement to investigate potential metabolism and ecological roles of microorganisms. Separation of intact cells from soil via FACS enabled mini-metagenomic sequencing, while bulk metagenomics provided total community context for benchmarking. Our approach generated 200 sorted-MAGs and 29 bulk metagenome MAGs of medium quality, expanding the known phylogenetic diversity (PD) of soil clades. Our data suggest that the sorted-MAGs represent some of the diversity of previously unsequenced organisms that are challenging to access using bulk approaches, offering insights into the functional potential of soil dark matter.

## RESULTS AND DISCUSSION

### Improved assembly and binning from mini-metagenomes.

**FIG 1:**
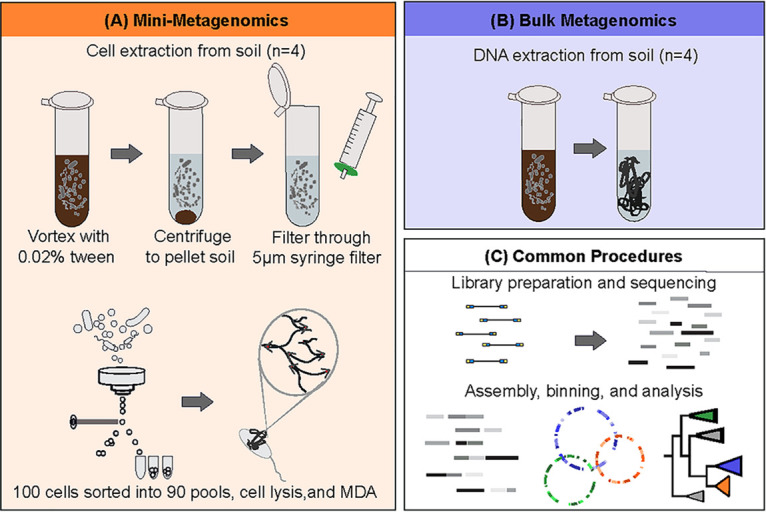
Overview of mini-metagenome and bulk metagenome approaches used in this study. (A) Mini-metagenomics performed on four soil samples, including one heated sample from the top organic soil, one heated sample from the lower mineral soil, one control organic sample, and one control mineral sample (*n* = 4). Cells were separated from soil particles using a mild detergent, followed by vortex mixing, centrifugation, and filtration through a 5-μm-pore-size syringe filter. Suspended cells were stained with SYBR green and sorted into 90 pools of 100 cells each, generating 359 mini-metagenomes. (B) Bulk metagenomic sequencing conducted on the four soils that were used in mini-metagenomics. (C) Following nucleic acid extraction, libraries were prepared, and shotgun sequencing was performed. Sequence data underwent assembly and quality control. Data were binned and assessed for bin quality. Only medium-quality genome bins with estimates of 50% completeness, 10% contamination, and 10% strain heterogeneity were used in downstream phylogenomic and functional analyses. Further details are provided in Materials and Methods.

Our application of mini-metagenomics combines microbial cell sorting and metagenome sequencing in order to divide a complex soil community into many smaller, less complex subsets. We performed FACS on pools of cells from four soil samples collected from the Barre Woods experimental warming plots at the Harvard Forest Long-Term Ecological Research (LTER) site. From each of the four samples we sequenced 90 replicate pools of 100 cells for a total of 359 mini-metagenomes (one mini-metagenome failed quality control standards). In conjunction with mini-metagenomic sequencing, we performed bulk metagenomics on these four soils, generating totals of 1.2 Gbp and 1.3 Gbp, respectively (Fig. 1; see also Table S1 in the supplemental material).

10.1128/mSystems.00768-19.7 TABLE S1 Sequencing summary statistics for sorted and bulk metagenomes. Download Table S1, CSV file, 0.03 MB. Copyright © 2020 Alteio et al. 2020 Alteio et al. This content is distributed under the terms of the Creative Commons Attribution 4.0 International license.

**FIG 2:**
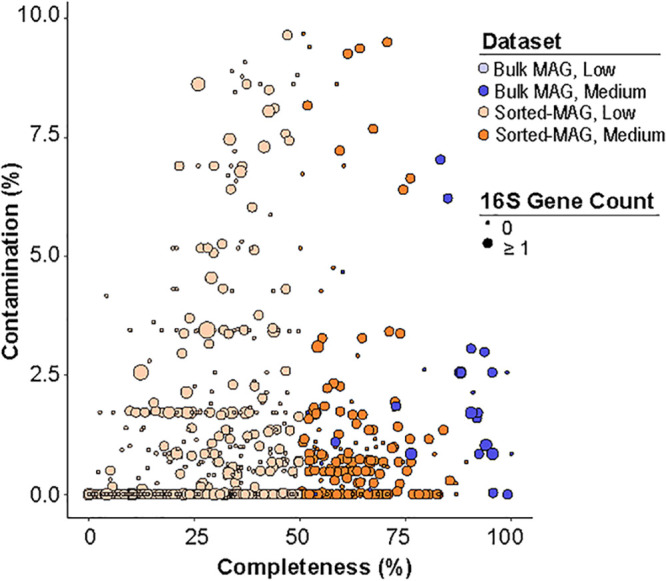
Assessment of sorted-MAG and MAG quality. Sorted-MAGs (orange, *n* = 1,793) and bulk MAGs from the four samples corresponding to those sorted with FACS (blue, *n* = 275) are represented. Medium-quality sorted-MAGs (dark orange, *n* = 200) and MAGs (dark blue, *n* = 29) are those with ≥50% completeness, ≤10% contamination, and ≤10% strain heterogeneity based on analysis of CheckM marker genes (43). The size of each circle represents the number of 16S rRNA gene copies within each MAG.

Binning of assembled contigs produced 1,793 mini-metagenome assembled genomes (sorted-MAGs) and 275 bulk metagenome MAGs (Fig. 2; see also Fig. S1 in the supplemental material). Following CheckM quality assessment (45), 200 sorted-MAGs and 29 bulk MAGs surpassed completeness thresholds of ≥50% complete, ≤10% contamination, and ≤10% strain heterogeneity. We considered MAGs with less than 50% completeness to represent “low quality” based on MIMAG standards (46) and excluded them from additional analyses (Fig. 2; see also Fig. S1). Overall, quality filtering removed lower-quality sorted-MAGs on the basis of completeness, whereas bulk MAGs were removed due to higher degrees of contamination and strain-level heterogeneity. Assessment of MAG quality using CheckM showed average percent completeness of 81.5% in medium-quality bulk metagenome MAGs (*n* = 29), which was higher than the 61.9% seen with the medium-quality sorted-MAGs (*n* = 200; *P* = 3.29 × 10^−7^) (Fig. 2; see also Fig. S1). Assessed for marker gene contamination, bulk metagenome MAGs revealed an average estimated level of contamination of 1.92%, indicating an estimated level of contamination higher than the average of 0.98% contamination in the sorted-MAGs (*P* = 0.01117) (Fig. 2; see also Fig. S1). Analysis of strain-level heterogeneity across medium-quality MAGs and sorted-MAGs revealed a lower degree of multiple strain contamination in sorted-MAGs than in bulk MAGs as assessed by CheckM (45). The average level of strain heterogeneity for the bulk MAGs was 1.16%, compared to 0.04% in the sorted-MAGs (*P* = 3.89 × 10^−6^; Table S2). This decrease in strain heterogeneity seen using mini-metagenomics indicates that sorted-MAGs collapse fewer strains into a single MAG.

10.1128/mSystems.00768-19.1 FIG S1 Summary of sequencing and MAGs. (A) Number of MAGs generated from mini-metagenomics and bulk metagenomic sequencing. Dark colored bars represent the total number of sorted-MAGs and bulk metagenome MAGs. Shaded bars represent medium-quality sorted-MAGs and bulk MAGs as determined by filtering at 50% completeness and 10% contamination based on CheckM marker genes (1). (B) Average GC composition for reads from mini-metagenome and bulk metagenome sequencing. Data represent percent GC in mini-metagenomes (*n* = 359) and bulk metagenomes (*n* = 4). The vertical center line represents the median GC percentage, with the lower and upper edges representing the first and third quartiles. Error bars represent standard deviations. (C to H) Quality assessment of assembly and binning from mini-metagenomes and bulk metagenomes. (C and F) Completeness and contamination of sorted-MAGs (C, orange) and bulk MAGs (F, blue) as assessed by CheckM marker genes. Low-completeness MAGs are less than 50% complete, and medium-completeness and high-completeness MAGs are at least 50% complete. MAGs at least 50% complete and less than 10% contaminated were retained in this study. (D and G) Number of scaffolds in medium-quality and high-quality sorted-MAGs (D, orange) and bulk MAGs (E, blue). (E and H) Counts of rRNA genes for 200 medium-quality sorted-MAGs (E, orange) and bulk MAGs (H, blue). Download FIG S1, PDF file, 0.1 MB. Copyright © 2020 Alteio et al. 2020 Alteio et al. This content is distributed under the terms of the Creative Commons Attribution 4.0 International license.

10.1128/mSystems.00768-19.8 TABLE S2 Sorted and bulk MAG completeness and contamination estimates from CheckM. Download Table S2, CSV file, 0.3 MB. Copyright © 2020 Alteio et al. 2020 Alteio et al. This content is distributed under the terms of the Creative Commons Attribution 4.0 International license.

As one measure to compare mini-metagenomics and bulk metagenomics methods, we assessed GC content and found averages of 49.2% GC and 60.5% GC in sorted-MAGs and MAGs, respectively (Fig. S1; see also Table S2). Variation in GC content can be attributed to known biases in the single-cell workflow such as susceptibility of cells to sorting and lysis (37, 47), as well as amplification bias introduced during MDA (48). The cell isolation method used in mini-metagenomics reduces inflation of community diversity as a result of exogenous DNA. Additionally, the difference in DNA extraction procedures between mini-metagenomics and bulk metagenomics represents an opportunity to capture an expanded diversity of microorganisms, as each approach may access a different component of the community. Taking the data together, mini-metagenomics and bulk metagenomics generated a large number of quality MAGs that can be used as complementary data sets in genome-resolved studies to investigate broad microbial diversity.

### Expansion of phylogenetic diversity.

**FIG 3:**
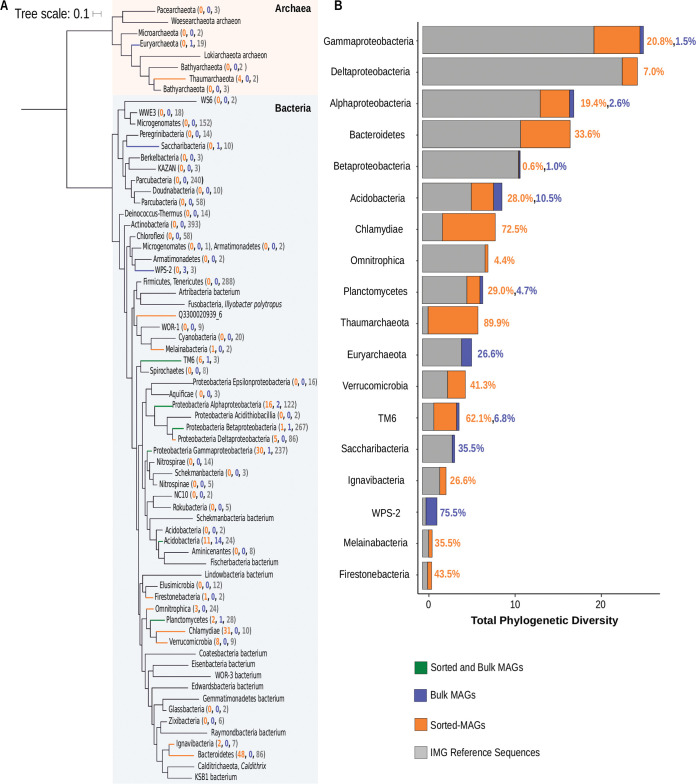
Phylogenetic diversity of soil taxa identified in this study. (A) Maximum likelihood tree of the phylogenetic distribution of medium-quality sorted-MAGs and bulk MAGs in the context of previously sequenced soil taxa. Colored branches represent clades that include sorted-MAGs and/or bulk MAGs. Orange branches include only sorted-MAGs, blue branches include only bulk MAGs, and green branches include both mini-metagenome and bulk MAGs. Numbers in orange represent numbers of contributed sorted-MAGs, blue numbers represent bulk MAGs, and gray numbers represent the number of reference sequences in each clade. (B) Phylogenetic diversity expansion through sorted-MAGs and bulk MAGs. Gray represents the total branch length contributed by soil reference sequences from the IMG database. Orange bars represent total branch length from sorted-MAGs, and blue represents branch length from bulk MAGs. The percentage of increase in phylogenetic diversity from this study is shown next to each bar.

As one aim of our study was to provide reference genomes that represent soil microbiome diversity, we evaluated the contribution of both sorted-MAGs and bulk MAGs to phylogenetic diversity in the context of previously published genomes of soil bacteria and archaea. We inferred the phylogenetic relationships using concatenated marker genes from the 200 sorted-MAGs, the 29 bulk MAGs, and 3,024 soil microbe reference genomes from the IMG/M (Fig. 3A) (21). For this analysis, we clustered sequences at 95% average nucleotide identity (ANI) to estimate distinct species-level lineages, resulting in 170 sorted-MAGs, 25 bulk MAGs, and 2,341 reference MAGs and isolate genomes from IMG/M (Fig. 3A; see also Fig. S2). This small decrease in the number of MAGs as a result of clustering indicates very little redundancy between previous MAGs and available reference sequences. Sorted and bulk MAGs from this study contributed genome diversity across numerous soil clades, including *Alphaproteobacteria* (16 sorted-MAGs, 2 bulk MAGs), *Acidobacteria* (11 sorted-MAGs, 14 bulk MAGs), and *Planctomycetes* (2 sorted-MAGs, 1 bulk MAG). Sorted and bulk MAGs also contributed diversity to less-abundant soil taxa, including *TM6* (6 sorted-MAGs, 1 bulk MAG) and *Betaproteobacteria* (3 sorted-MAGs, 1 bulk MAG).

**FIG 4:**
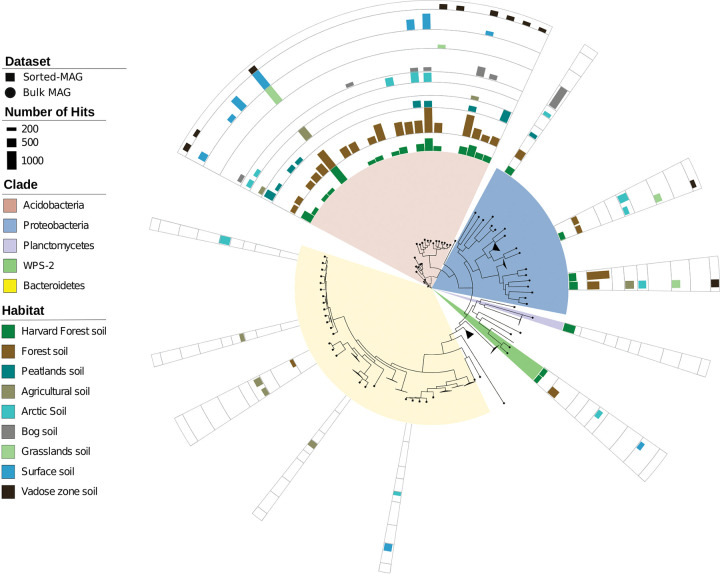
Comparison of MAGs from this study with published data from terrestrial metagenomes. Innermost is a maximum likelihood tree based on a concatenated alignment of 56 conserved marker proteins from medium-quality sorted-MAGs and bulk MAGs recovered in this study. Mini-metagenomes and bulk MAGs were dereplicated by clustering at 95% average nucleotide identity, resulting in 173 sorted-MAGs and 28 bulk MAGs. The clade names are color-coded according to phylum. Individual tracks around the tree depict hits of individual sorted-MAGs and bulk MAGs by metagenome samples arising from each terrestrial habitat type as specified in the legend. The height of the bar chart indicates the total number of sorted-MAGs and bulk MAG coding sequences that matched metagenome samples. The MAGs were considered matches if they had a minimum of 200 coding sequences with hits at ≥95% amino acid identity over 70% alignment lengths to CDS of an individual metagenome. Further details are provided in Materials and Methods, and data corresponding to this figure are provided in Table S3. The figure was rendered using iTOL (96).

10.1128/mSystems.00768-19.2 FIG S2 Heat maps of average nucleotide identity (ANI) clustering. (A) Heat map of ANI clustering from sorted-MAGs and MAGs in phylogenetic tree analysis. A set of 3,024 reference sequences from IMG/M and 200 sorted-MAGs and 29 bulk MAGs were clustered at 95% average nucleotide identity prior to phylogenetic tree construction (Fig. 3). Clustering resulted in 2,341 reference sequences, 170 sorted-MAGs, and 25 bulk MAGs for tree construction. Trees depict the 3,024 sorted-MAGs, bulk MAGs, and references. Sequence comparisons with low similarity are shown in red, and those with high similarity are shown in yellow. (B) Heat map of ANI clustering for sorted-MAGs and MAGs in metagenome recruitment analysis. The set of 200 medium-quality sorted-MAGs and the set of 29 medium-quality bulk MAGs were clustered at 95% ANI prior to metagenome recruitment (Fig. 4). The best representative of each cluster was selected for metagenome recruitment analysis, resulting in 173 sorted-MAGs and 28 bulk MAGs. Sequence comparisons with low similarity are shown in red, and those with high similarity are shown in yellow. Download FIG S2, PDF file, 1.1 MB. Copyright © 2020 Alteio et al. 2020 Alteio et al. This content is distributed under the terms of the Creative Commons Attribution 4.0 International license.

Comparison of MAGs recovered through mini-metagenome and bulk metagenomics revealed a broad diversity of soil bacteria, as well as a few archaeal taxa, and demonstrated the complementarity of these approaches for biological discovery. The sorted-MAGs expanded the known diversity of the taxa which were previously found to be abundant and ubiquitous across soil types (49), as well as of the taxa considered part of the rare biosphere that may still be widespread but remain at relatively low abundances in microbial communities (33). The more abundant taxa represented by the sorted-MAGs include *Bacteroidetes* (*n* = 48) and *Verrucomicrobia* (*n* = 8), while the taxa with typically lower abundances in soils included *Thaumarchaeota* (*n* = 4), *Omnitrophica* (*n* = 3), *Ignavibacteria* (*n* = 2), *Melainabacteria* (*n* = 1), and *Firestonebacteria* (*n* = 1) (1, 49). Interestingly, numerous sorted-MAGs belonged to phyla typically comprised of pathogens and endosymbionts such as the *Chlamydiae* (*n* = 31) and *Gammaproteobacteria*, specifically within the order *Legionellales* (*n* = 30), as well as *TM6* (*n* = 7) (50–53) (Fig. 3A; see also Fig. S3). Genomes in the phylum *Chlamydiae* and in the order *Legionellales* within the phylum *Gammaproteobacteria* are considered entirely intracellular (54, 55). The phyla identified by sorted-MAGs represented abundant taxa found in previous soil community studies (1, 49, 56) in addition to the rare biosphere, demonstrating the utility of mini-metagenomics for expanding diversity beyond abundant soil taxa (Fig. 3; see also Fig. S3).

10.1128/mSystems.00768-19.3 FIG S3 Relative abundance of taxa across sorted-MAGs, unsorted MAGs, and unbinned metagenomes. (A) Relative abundances of taxa across omics approaches. Taxonomy was assigned using blastp searches against the NCBI NR database, implemented in DIAMOND (2). Taxonomic classification was assessed using MEGAN (3), and relative abundances of assigned taxa were visualized in R using ggplot2 (4). (B) Taxonomic distribution of sorted-MAGs and MAGs using CheckM lineage-specific markers. Binning from mini-metagenomics (*n* = 359) captured broader taxonomic diversity than did binning from bulk metagenomic data (*n* = 4). Taxa where no substantial contribution to phylogenetic diversity was made (Fig. 3) may have representative MAGs that did not surpass the quality filtering threshold (≥50% completeness, ≤10% contaminated, and ≤10% strain heterogeneity). Download FIG S3, PDF file, 0.1 MB. Copyright © 2020 Alteio et al. 2020 Alteio et al. This content is distributed under the terms of the Creative Commons Attribution 4.0 International license.

As for the bulk MAGs, some of these belonged to rare taxa not recovered through mini-metagenomics, including *WPS-2* (*n* = 3), *Euryarchaeota* (*n* = 1), and *Saccharibacteria* (*n* = 1). We assessed phylogenetic diversity (PD), the total amount of branch length contributed by sequences of interest within a phylogenetic tree, in the sorted-MAGs to determine the contribution of this single study to the known range of microbial diversity. Calculation of phylogenetic diversity revealed a 7.2% increase in total branch length contributed by the sorted-MAGs in relation to the soil reference sequences from IMG/M (Fig. 3B). Mini-metagenomes expanded the range of available evidence not only of phylogenetic diversity within clades of known soil bacteria and archaea but also of candidate phyla and low-abundance taxa typically found in forest soils. More specifically, the sorted-MAGs increased the branch lengths of well-studied bacterial groups, including *Bacteroidetes* (33.6%) and *Alphaproteobacteria* (19.4%), along with those of groups notoriously recalcitrant to laboratory cultivation, such as *TM6* (62.1%), *Verrucomicrobia* (41.3%), and *Acidobacteria* (28.0%) (42, 57). Most notable was the PD increase in the *Chlamydiae* (72.5%), a taxonomic group which is typically overlooked in soil metagenomic studies due to their low abundance and likely dependence on eukaryotic host cells (58). We hypothesize that the application of mild detergent and syringe filtration during sample processing may have lysed the microbial eukaryotes that serve as hosts for bacterial endosymbionts, making these bacteria more accessible for FACS. A similar phenomenon was suggested for the detection of 16 novel giant viruses from these same samples (40), as these viruses are most often associated with eukaryotic host cells (59). The hypothesized liberation of these intracellular bacteria makes mini-metagenomic sequencing a useful tool for investigating the diversity and evolution of the intracellular life strategy (55, 60).

The sorted-MAGs demonstrated the potential for mini-metagenomics to increase our knowledge of diversity beyond what can be achieved using MAGs from bulk metagenome studies alone. The bulk MAGs contributed to the phylogenetic diversity of many of the same clades of soil bacteria as the sorted-MAGs, including *Acidobacteria* (10.5%), *TM6* (6.8%), and *Alphaproteobacteria* (2.6%). Even in clades where more bulk-derived genomes were added than sorted-MAGs, such as in *Acidobacteria*, the sorted-MAGs were phylogenetically more diverse. These calculated increases in phylogenetic diversity with the addition of MAGs from this study are limited with regard to scope, as not all available reference sequences are publicly accessible in the IMG/M database. However, this database is updated monthly with newly uploaded sequences from GenBank (21).

### Complementarity of mini-metagenomics and bulk metagenome sequencing.

Mini-metagenomics has not been widely applied in soils to date and will serve as a valuable tool for expanding our knowledge of soil biodiversity. In this study, we applied both bulk metagenomics and mini-metagenomics to compare analyses of complex community samples as well as to identify the advantages and disadvantages of each. This approach is capable of generating higher-quality MAGs than bulk metagenomics due to the reduction of strain-level microheterogeneity when selected pools of cells are sequenced (32). Although they are lower in estimated genome completeness than bulk MAGs, sorted-MAGs from soil also demonstrate a lower degree of strain heterogeneity, indicating that fewer genomic fragments from multiple organisms have been collapsed into a single genome bin (45) (Table S2). The sorted-MAG reduced genome completeness is, at least in part, a likely result of uneven whole-genome amplification (WGA), as has been extensively reported in single-cell genomic studies (47). The larger number of sorted-MAGs presents opportunities for improved resolution for taxonomic classification and for genome-informed investigations of microbial metabolism and linking the potential metabolism to processes at the ecosystem level. Taxonomic classification of organisms using high-quality MAGs has become a critical approach for expanding knowledge of microbial diversity, given that we currently lack information for the majority of uncultivated organisms (61). Finally, although not applied in this study, FACS-based sample processing may be modified to achieve cell and/or population separation that is more highly targeted (62), thereby further expanding the utility of mini-metagenomics to detect microbial dark matter.

Although the mini-metagenomics approach produced a greater number of medium-quality genome bins than bulk metagenomics, this approach is not without challenges. In comparison to bulk metagenomics, the requirements associated with mini-metagenomics may be prohibitive, as it involves equipment and expertise that may not be easily accessible. In addition to logistical obstacles, methodological challenges, including cell isolation and GC-based genome amplification skew, likely introduce bias during sample processing. The formation of extracellular polysaccharides is a strategy widely used by microorganisms to protect against changes in the environment, as well as for exchange of nutrients and materials (63). These matrices may support the maintenance of stable microbial consortia and cellular adhesion to soil particles (63). These larger aggregate structures are subject to exclusion in sample preparation steps, including filtration, prior to FACS. Methodological challenges such as these may be reflected in our data, where organisms which are typically abundant in forest soils, such as *Actinobacteria*, *Chloroflexi*, and *Firmicutes* (49), were present in low numbers using mini-metagenomics compared to traditional bulk metagenomics (Fig. 3; see also Fig. S3). Though these taxa might have been missed due to the aforementioned biases, it is also possible that sequences from these organisms were not binned or were placed in a lower-quality bin based on our filtering threshold. For example, bacteria in the phylum *Spirochaetes* were represented by 47 distinct sorted-MAGs; however, none of these passed quality filtering standards and all were therefore excluded (Fig. 3; see also Fig. S3). An alternative DNA amplification method, termed WGA-X, has been developed which improves cell lysis and amplification of high-GC-content organisms over MDA (48). With this improved method of DNA amplification, more extensively representative mini-metagenomic sampling might be possible.

Bulk metagenomics presents fewer opportunities to introduce bias and may more accurately capture the total soil community than the mini-metagenomic approach. Using bulk metagenomics, DNA from the total soil sample is extracted and sequenced, which circumvents cell and particle size selection introduced via FACS. Thus, bulk metagenomics remains an invaluable tool for understanding the diversity of microbial communities, particularly that of the dominant soil microorganisms. Sorted-MAGs, however, provided additional genomic data covering broader phylogenetic diversity compared to the bulk MAGs, further enhancing biological discovery. The scientific question of interest should guide the selection of one approach over the other. We support the use of both approaches in complement to one another in order to capture the broadest scope of soil microbial diversity.

### Representation of sorted-MAGs and MAGs across terrestrial soil metagenomes.

To assess the representation of our newly generated soil reference genomes across other terrestrial ecosystems, we searched for protein coding sequences from our collection of sorted-MAGs and MAGs across publicly available soil metagenomes from 80 terrestrial metagenome studies. For this analysis, we dereplicated the 200 sorted-MAGs and 29 bulk MAGs from this study by clustering at 95% average nucleotide identity without reference sequences, resulting in 173 sorted-MAGs and 28 bulk MAGs as cluster representatives (Fig. 4; see also Fig. S2). We assessed these sorted-MAGs and bulk MAGs in the context of broader terrestrial community studies by comparing them against 2,210 metagenomes from the 80 terrestrial studies using LAST (64) (Fig. 4; see also Table S3). We defined highly represented sorted-MAGs and MAGs as those with at least 200 protein coding sequences with hits to metagenome samples at ≥95% amino acid identity (AAI) over a 70% alignment length (65, 66).

10.1128/mSystems.00768-19.9 TABLE S3 Selected terrestrial metagenomes publicly available on IMG/M. Download Table S3, CSV file, 0.7 MB. Copyright © 2020 Alteio et al. 2020 Alteio et al. This content is distributed under the terms of the Creative Commons Attribution 4.0 International license.

Some of our sorted-MAGs and MAGs detected in previous metagenomic soil investigations were members of the phylum *Acidobacteria* (10 sorted-MAGs and 15 MAGs) (Fig. 4; see also Table S3). Five bulk MAGs in the phylum *Proteobacteria* were detected in metagenomes from forest, agricultural, arctic, grassland, and vadose zone soils, whereas two bulk MAGs in candidate division *WPS-2* were detected in metagenomes from Harvard Forest and other forest soil metagenomes, as well as arctic and surface soils. Interestingly, one MAG in the *Planctomycetes* was detected only in metagenome sequences from the Harvard Forest, indicating that this may represent a unique MAG which had not been found in previous terrestrial metagenome studies.

The phylum *Acidobacteria* was the most abundant phylum represented in the bulk MAGs (77%) and unbinned metagenome data (32%), compared to the sorted-MAGs (8.5%) (Fig. S3). In contrast, the phylum *Bacteroidetes* was highly represented by the sorted-MAGs (55.5%), compared to the bulk metagenome MAGs (0.1%) and unbinned metagenome data (3.8%) (Fig. S3). The sorted-MAGs in the phylum *Bacteroidetes* increased the phylogenetic diversity of this group by 33.6% (Fig. 3) and appeared to be novel as they had a relatively low number of matches to protein coding sequences from publicly available soil metagenomes, with only 6 of 67 *Bacteroidetes* MAGs having similarity of at least 200 coding sequences with published soil metagenomes (Fig. 4). This presumed novelty could also contribute to computation challenges associated with sequence assembly, as only the most abundant taxa are overrepresented in public databases (29). And yet many of these sorted and bulk MAGs were not represented in previous Harvard Forest metagenomes (Fig. 3). Taking the data together, the low level of representation of our *Bacteroidetes* sorted-MAGs across previously published metagenome samples illustrates the expanded biodiversity gained through the use of mini-metagenomes, demonstrating the utility of this approach for accessing the rare taxa within phylogenetically diverse samples.

### Biological insights into carbon metabolism in soil *Bacteroidetes*.

**FIG 5:**
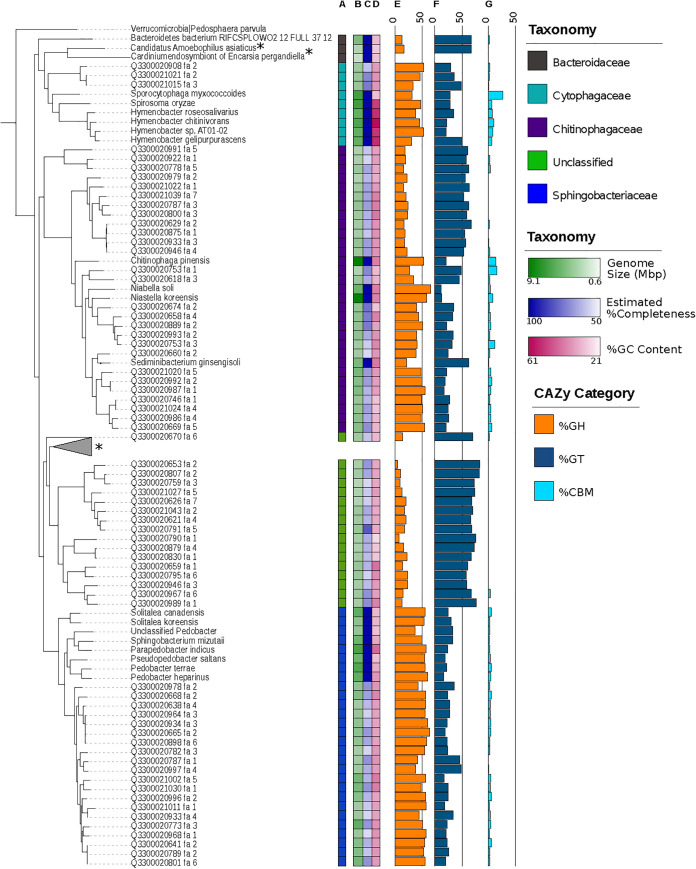
Insights into carbon metabolism within the phylum *Bacteroidetes*. A concatenated marker gene tree of 67 *Bacteroidetes* sorted-MAGs and 70 *Bacteroidetes* reference sequences from the IMG/M database shows clade-specific abundances of glycoside hydrolase and glycosyl transferases. The tree is rooted with Pedosphaera parvula (phylum *Verrucomicrobia*). Column A shows the distribution of sorted-MAGs across three families of *Bacteroidetes*, including *Cytophagaceae*, *Chitinophagaceae*, and *Sphingobacteriaceae*, and a clade of unclassified sorted-MAGs. Column B shows genome sizes, with the darkest color representing the largest genome of 9.1 megabases and the lightest representing a genome size of 0.6 megabases. Column C shows genome completeness based on CheckM marker genes, ranging from 50% to 80.5%, as a color gradient. Reference sequences represent isolates with complete genomes. Column D presents genome GC content as a color gradient that ranges from 21.13% to 61.24%. In columns E to G, percentages of genes annotated as glycoside hydrolases (column E), glycosyl transferases (column F), and carbohydrate binding modules (column G) are illustrated as bar charts with vertical lines denoting 0% and 50% of annotated genes. *Bacteroidetes* with known symbiotic relationships are indicated with an asterisk. The collapsed clade contains Sulcia muelleri, a known symbiont of sap-feeding insects, and *Blatellabacterium* sp., a known symbiont of the cockroach Blatella germanica.

*Bacteroidetes* spp. make up ∼10% to the total microbial community in soils (1), and yet most of our knowledge about members of this phylum stems from sequenced isolates from vertebrate guts and aquatic habitats (67–69). Bacteria in the phylum *Bacteroidetes* are known to be important degraders of polysaccharides; however, little is known about the role of this abundant group in soils. Given the relatively small body of work on soil *Bacteroidetes* and the substantial contribution of 67 putatively novel sorted-MAGs from this study to phylogenetic diversity estimates (Fig. 3; see also Fig. 5), we further explored these sorted-MAGs from *Bacteroidetes* to gain insight into their physiological potential and assess functional similarities to previously known *Bacteroidetes*.

The genome sizes of the sorted-MAGs ranged from 1.6 to 5 Mb (Table S4), which is within the range of previously reported *Bacteroidetes* genome sizes of from 0.9 Mb (*Cardinium* endosymbiont) (70) to 9.1 Mb (Chitinophaga pinensis) (71). The finding of smaller genome sizes of the sorted-MAGs was likely due to genome completeness estimates, which ranged from 50% to 80.5% based on analysis of CheckM marker genes (Fig. 5; see also Fig. S4) (45). These sorted-MAGs were distributed across three distinct families, including *Cytophagaceae*, *Chitinophagaceae*, and *Sphingobacteriaceae*, as well as a clade of unclassified sorted-MAGs (Fig. 5). *Bacteroidetes* are known to have a large set of genes that encode enzymes for carbohydrate degradation (69), including a broad array of glycoside hydrolases that are phylogenetically conserved (72). The distribution of CAZy gene families across these *Bacteroidetes* taxa exhibited clade-specific abundance patterns of glycoside hydrolases, glycosyl transferases, and carbohydrate binding modules (Fig. 5; see also Table S4) (73).

10.1128/mSystems.00768-19.4 FIG S4 Summary of *Bacteroidetes* MAGs. (A) Quality assessment of *Bacteroidetes* sorted-MAGs. Data represent completeness and contamination of sorted-MAGs. Gray points represent all 1,793 sorted-MAGs and 275 bulk MAGs prior to quality filtering. Magenta points represent 67 medium-quality sorted-MAGs within the phylum *Bacteroidetes*. Point size refers to the number of 16S rRNA genes within the sorted-MAGs. (B) Relationship between genome size and number of CAZymes in *Bacteroidetes* sorted-MAGs. The number of CAZy genes in each of the six families (5), including auxiliary activities (AA), glycoside hydrolases (GH), carbohydrate binding modules (CBMs), carbohydrate esterases (CE), polysaccharide lyases (PL), and glycosyl transferases (GT), is plotted as a factor of sorted-MAG size in megabases. Download FIG S4, PDF file, 0.2 MB. Copyright © 2020 Alteio et al. 2020 Alteio et al. This content is distributed under the terms of the Creative Commons Attribution 4.0 International license.

10.1128/mSystems.00768-19.10 TABLE S4 Representation of sorted-MAGs and bulk MAGs across terrestrial metagenome studies. Download Table S4, CSV file, 0.3 MB. Copyright © 2020 Alteio et al. 2020 Alteio et al. This content is distributed under the terms of the Creative Commons Attribution 4.0 International license.

Sorted-MAGs within the *Cytophagaceae* family appeared to be specialized for polymeric carbon degradation, namely, degradation of cellulose, as they encode proteins in glycoside hydrolase family 5 which exhibit endocellulase activity (74, 75). In contrast, members of the *Chitinophagaceae* and *Sphingobacteriaceae* families appeared to be generalists in carbon utilization. More specifically, the *Chitinophagaceae* sorted-MAGs harbored the potential to use cellulose, hemicellulose, and chitin. Seventeen of the 27 sorted-MAGs in the *Chitinophagaceae* family contained at least one chitinase in glycoside hydrolase family 18 or 19 (76) along with cellulases in glycoside hydrolase families 5, 8, and 9 and glycoside hydrolases in family 43 that may degrade hemicellulose and pectin (77) (Fig. 5; see also Fig. S5). In support of this conjecture, the sequenced genome of Chitinophaga pinensis (a member of the *Chitinophagaceae* family) contains genes to degrade leaf matter and fungal structures, suggesting its ability to degrade both cellulose and chitin (78). Twenty sorted-MAGs belonged to the family *Sphingobacteriaceae* and typically harbored the potential to degrade cellulose, xylan, and chitin, with GH families 2, 3, 5, 13, 18, and 20 being the most abundant across sorted-MAGs in this group. Interestingly, one sorted-MAG (Q3300020668_2) had the highest number of glycoside hydrolase genes within the *Sphingobacteriaceae* (125 annotated glycoside hydrolases), representing a diverse array of carbohydrate degradation capabilities and potential metabolic flexibility. This is consistent with previous investigations describing the family *Sphingobacteriaceae* as capable of degrading diverse polysaccharides (79).

10.1128/mSystems.00768-19.5 FIG S5 Distribution of CAZymes across *Bacteroidetes* sorted-MAGs. A presence/absence heat map of CAZy genes across the 67 *Bacteroidetes* sorted-MAGs from this study is shown. The sorted-MAGs reveal a clade-specific pattern of CAZyme distribution, with many sorted-MAGs in the unclassified clade lacking many glycoside hydrolase genes for polysaccharide degradation. Download FIG S5, PDF file, 2.3 MB. Copyright © 2020 Alteio et al. 2020 Alteio et al. This content is distributed under the terms of the Creative Commons Attribution 4.0 International license.

Putatively novel *Bacteroidetes* sorted-MAGs stemming from experimental warming plots at the Harvard Forest Long-Term Ecological Research site spanned three different taxonomic families and harbored an extensive diversity of enzyme families, including those involved in hydrolysis of polymeric chitin, cellulose, and hemicellulose substrates. The genomic potential to utilize these labile carbon compounds is consistent with previous metagenomic investigations in soils of warmed plots (16, 80). Interestingly, the number of identified carbohydrate active enzyme genes increased with genome size for each of the six CAZy categories (Fig. 5; see also Fig. S4), illustrating that these organisms accumulated the capacity to degrade various carbohydrates, thereby expanding their niche for carbohydrate utilization in soil. And yet 17 sorted-MAGs belonged to an unclassified clade of *Bacteroidetes* spp. which were depleted in glycoside hydrolases and carbohydrate binding modules but retained a high number of glycosyl transferases (Fig. 5; see also Fig. S5), suggesting a limited role for these organisms in substrate decomposition. Rather, the relatively higher abundance of glycosyl transferase genes involved in the formation of glycosidic bonds may indicate that these organisms are responsible for synthesis of higher-molecular-weight compounds and may depend on living in close association with other organisms.

To further support the role of the *Bacteroidetes* in polymeric carbon degradation in soils, we investigated specific carbohydrate degradation using the KEGG database (81, 82) and predicted the completeness of metabolic pathways using KEGG-Decoder (83). The majority of sorted-MAGs in *Sphingobacteriaceae* and *Chitinophagaceae* have nearly complete pathways coding for alpha-amylase, beta-glucosidase, chitinase, and diacetylchitobiose deacetylase activity, further supporting the idea of a role of these organisms as generalists in polysaccharide degradation (Fig. S6). Additionally, seven of the sorted-MAGs within *Sphingobacteriaceae* contain nearly complete pathways for pullulanase. Consistent with analysis of carbohydrate degradation potential using the CAZy database (Fig. S5), 22 of the sorted-MAGs were found to contain only one complete pathway or no complete pathways for polymeric carbohydrate degradation (Fig. S6). This limited potential for carbohydrate utilization does not correlate with decreased genome completeness (Fig. S4). Rather, we hypothesize that these sorted-MAGs have an alternative survival strategy in the soil environment similar to those exhibited by other *Bacteroidetes*, including “*Candidatus* Amoebophilus asiaticus” (84), *Cardinium* sp. (85), “*Candidatus* Sulcia muelleri” (86), and *Blattabacterium* sp. (87), which are known symbionts (Fig. 5).

10.1128/mSystems.00768-19.6 FIG S6 Completeness of KEGG pathways across *Bacteroidetes* sorted-MAGs. Completeness of KEGG metabolic pathways was assigned using KEGG-Decoder (6, 7) for the 67 *Bacteroidetes* sorted-MAGs. The legend displays the percentage of completeness of each pathway or process, with incomplete pathways/processes indicated in light yellow and complete pathways/processes in dark red. Download FIG S6, PDF file, 2.3 MB. Copyright © 2020 Alteio et al. 2020 Alteio et al. This content is distributed under the terms of the Creative Commons Attribution 4.0 International license.

Similarly to known symbionts, the estimated GC contents of unclassified sorted-MAGs in this study were low relative to those of other *Bacteroidetes* sequences, with an average of 39.97% GC (88). These unclassified *Bacteroidetes* demonstrate limited ability for carbon utilization and reduced central carbon metabolism and chemotaxis (Fig. S6) while retaining genome sizes of 2.4 Mb on average, which are comparable to those of *Bacteroidetes* previously identified as host-associated species (Fig. 5; see also Fig. S5 and S6). Symbionts may undergo the process of reduction in genome size when in contact with the host organism, resulting in a linear relationship between the number of protein coding genes contained and the size of the genome (87–89). The abundance of unclassified *Bacteroidetes* within this study may represent further evidence of the liberation of symbionts from host cells and vacuoles prior to FACS. Alternatively, the relatively low carbohydrate degradation potential of sorted-MAGs within the unclassified clade may be indicative of an opportunistic life strategy (74).

### Conclusions.

This application of mini-metagenomics and bulk metagenomics has demonstrated the utility of these complementary techniques for biological discovery within the complex soil ecosystem. Using mini-metagenomics to reduce the number of cells prior to sequencing, we uncovered bacterial and archaeal soil diversity that could not be accessed using bulk metagenomics alone. Mini-metagenomics is a powerful tool for the discovery of rare biosphere organisms and potential endosymbionts, revealing biodiversity in dominant soil groups as well as in low-abundance taxa. Taken together, the mini-metagenomics and bulk metagenomics approaches allow us to probe deeper into microbial diversity and function within heterogeneous environments beyond soil.

## MATERIALS AND METHODS

### Sample collection and incubation.

Soils were collected on the 24th of May 2017 from the Barre Woods long-term experimental warming plots located at the Harvard Forest Long Term Ecological Research (LTER) site in Petersham, MA, USA. This site consists of two 30-by-30-m plots: one which has remained at ambient soil temperature and one that has been artificially warmed since 2002 using heating cables buried at 10-cm depth (90). Soil respiration, nitrogen mineralization, and vegetation cover and growth as well as soil and litter chemistry have been measured over the course of the long-term experiment. The canopy overstory is dominated by paper birch and black birch (Betula papyrifera and B. lenta, respectively), red maple (Acer rubrum), black oak and red oak (Quercus velutina and Q. rubra, respectively), and American beech (Fagus grandifolia) (56).

Two intact soil cores were taken from subplots within the larger 30-by-30-m experimental plots, including a subplot within heated plot 2 and a subplot within control plot 12. The subplots included in this study were chosen at random. The collected soil cores were separated into organic (approximately top 5 cm of soil core) and mineral (lower 5 cm of soil core) horizons by visual inspection and were sieved with a 2-mm-pore-size mesh, resulting in a total of 4 individual soil samples.

Both treatments (heated and control) and soil horizons (organic and mineral) were represented by these four soil samples. Approximately 5 g of soil was immediately frozen in a dry ice/ethanol bath for DNA extraction and was then transported to the University of Massachusetts Amherst for storage at –80°C. Approximately 15 g of soil was transferred to a 50-ml Falcon tube for transportation on ice to the Joint Genome Institute (JGI) in Walnut Creek, CA, USA. Samples were further processed as described previously Schulz et al. (40). The study was limited to four soil samples in order to maintain the cost-effectiveness and overall efficiency of the techniques applied.

### Sample preparation and cell sorting.

Cells were separated from four incubated soils (heated organic, heated mineral, control organic, and control mineral samples) for FACS through the addition of 0.02% Tween 20 followed by vortex mixing performed for 5 min. Samples were centrifuged for 5 min at 500 × *g* to pellet large soil particles. Following centrifugation, the supernatant was filtered through a 5-μm-pore-size syringe filter to remove the remaining soil particulates. Samples were diluted 1:100 in phosphate-buffered saline (PBS) and stained with SYBR green (Thermo Fisher Scientific, Waltham, MA, USA). For each of the four soil samples, 90 pools of 100 SYBR-positive (SYBR^+^) cells were sorted into microwell plates using a BD Influx cell sorter (BD Biosciences, San Jose, CA, USA) to perform FACS. Sorted pools underwent cell lysis and whole-genome amplification using a Qiagen RepliG single-cell kit for multiple-displacement amplification (MDA) (Qiagen, Hilden, Germany). A total of 360 libraries were generated for sequencing with a Nextera XT v2 kit (Illumina, San Diego, CA, USA) with 9 rounds of PCR amplification.

### Mini-metagenomes.

Following library preparation, the 360 mini-metagenome libraries were sequenced on an Illumina NextSeq platform (Illumina, San Diego, CA, USA) at the DOE Joint Genome Institute (JGI; Walnut Creek, CA, USA). Pools of 90 libraries were processed in four sequencing runs with 2 × 150-bp read lengths. Raw Illumina reads were quality filtered to remove contamination and low-quality reads using BBTools (v37.38) (91), resulting in 359 mini-metagenomes for downstream analysis, as one mini-metagenome did not pass quality filtering standards. Read normalization was performed using BBNorm (91), and error correction was conducted using Tadpole (91). Assembly of filtered, normalized Illumina reads was completed using SPAdes (v3.10.1) (92) with the following options: –phred-offset 33 -t 16 -m 115 –sc -k 25,55,95. All contig ends were trimmed of 200 bp, and contigs were discarded if the length was <2 kb or the level of read coverage was less than 2 using BBMap (91) with the following options: nodisk ambig, filterbycoverage.sh: mincov.

### Bulk metagenomes.

Total DNA was extracted from ∼0.25 g of soil using a DNeasy PowerSoil DNA extraction kit (Qiagen, Hilden, Germany). Extracted DNA was assessed using a Bioanalyzer (Agilent Technologies, Santa Clara, CA, USA) and Qubit (Thermo Fisher Scientific, Waltham, MA, USA). Unamplified TruSeq libraries were prepared for 4 DNA samples prior to sequencing on an Illumina HiSeq-2000 platform (Illumina, San Diego, CA, USA) at the DOE JGI. Raw Illumina reads were trimmed, quality filtered, and corrected using bfc (version r181) with the following options: -1 -s 10g -k 21 -t 10. Following quality filtering, reads were assembled using SPAdes (v3.11.1) (92) with the following options: -m 2000 –only-assembler -k 33,55,77,99,127 –meta -t 32. The entire filtered read set was mapped to the final assembly, and coverage information was generated using BBMap (v37.62) (91) with default parameters except ambiguous=random. The version of the processing pipeline used was jgi_mga_meta_rqc.py, 2.1.0. Of the 28 metagenome samples sequenced, only 4 were selected for inclusion in analysis for this study because they corresponded to those samples sorted using FACS.

### Genome binning and quality assessment.

Assembled contigs from the bulk and mini-metagenomes were binned into MAGs and sorted-MAGs based on tetranucleotide frequency using MetaBat2 (93). Sorted-MAGs were generated for mini-metagenomes without contig coverage patterns due to MDA bias. Genome bins were assessed for estimated completeness and estimated contamination marker genes included in the CheckM (45). Bulk metagenome MAGs and sorted-MAGs were filtered to ≥50% completeness, ≤10% contamination, and ≤10% strain heterogeneity to retain medium-quality sorted-MAGs and bulk metagenome MAGs for downstream analysis (46). Following quality filtering, 200 medium-quality sorted-MAGs and 29 medium-quality bulk metagenome MAGs were used for phylogenomic analysis, metagenomic recruitment, and investigation of metabolic potential.

### Phylogenetic tree construction and phylogenetic diversity.

A concatenated marker gene phylogenetic tree was constructed for 200 medium-quality sorted-MAGs, 29 bulk MAGs, and 3,024 reference genomes from soil bacteria and archaea available in the IMG/M database. A set of 56 universal single-copy marker proteins (41, 92) was identified with hmmsearch (v3.1b2) (94) and specific hidden Markov models (HMMs) for each of the markers. For every marker protein, alignments were built with MAFFT (v7.294b) (95) and subsequently trimmed with BMGE using BLOSUM30 (96). MAGs and reference sequences were clustered at 95% average nucleotide identity with FastANI v1.0 (97), resulting in 170 sorted-MAGs, 25 bulk MAGs, and 2,341 reference sequences with distinct taxonomic classifications. Single-protein alignments were then concatenated, and a phylogenetic tree was inferred with FastTree2 using the options -spr 4 -mlacc 2 -slownni -lg (98) and was visualized using iTol (99).

The contribution of sorted-MAGs and bulk MAGs to phylogenetic diversity was determined by calculating the sum of the total branch lengths of the contributed genomes relative to the reference genomes (100). Total branch length was calculated for a phylogenetic tree containing only 2,341 bacterial and archaeal reference sequences from IMG/M (21). We then calculated the additional total branch lengths contributed by sorted-MAGs and bulk MAGs. The percentage of increase in total branch length was determined for the complete phylogenetic tree, as well as for clades that included sorted-MAGs.

Taxonomy was assigned to sorted-MAGs, bulk MAGs, and metagenome reads by searching sequences against the NCBI-NR database using DIAMOND (101). BLAST results were imported into MEGAN6 (102) for taxonomic assignment. The relative abundance of each phylum was computed and visualized in R using ggplot2 (103).

### Protein recruitment.

Sorted-MAGs (*n* = 200) and bulk MAGs (*n* = 29) were dereplicated by clustering based on 95% average nucleotide identity. Protein coding sequences from the resulting 199 representative sorted-MAGs and MAGs were compared against coding sequences predicted from 2,210 soil metagenome samples from 80 terrestrial metagenome studies stored in the IMG/M database using LAST (64) (Fig. 4; see also Table S3 in the supplemental material). Individual sorted-MAGs and MAGs were designated a match to metagenome samples if the following criteria were met: a minimum of 200 coding DNA sequences (CDS) with hits at ≥ 95% amino acid identity over 70% alignment lengths to CDS of an individual metagenome. The rationale for choosing the minimum 200 hit count was to ensure that the evidence included more than merely housekeeping genes, which may be more highly conserved. The 95% amino acid identity cutoff was chosen based on a study reported previously by Luo et al. (65), who asserted that organisms grouped at the “species” level typically show >85% AAI among themselves. Since our data set included divergent sublineages, the more conservative threshold of 95% amino acid identity was adopted. The average percentage of CDS with a metagenome hit was calculated for each mini-metagenome (Fig. 4; see also Table S4), and the results were plotted as a multibar chart in iTol (99).

### *Bacteroidetes* phylogeny and metabolic predictions.

A maximum likelihood tree for *Bacteroidetes* was constructed using IQTree (104) for the 67 sorted-MAGs and soil *Bacteroidetes* references from IMG/M. The tree was rooted with Pedosphaera parvula in the phylum *Verrucomicrobia*. Family-level taxonomic classification and genome size and genome size based on CheckM marker gene assessment (45) were visualized using iTol (99). Functional annotation for sorted-MAGs was assigned using the Carbohydrate Active Enzyme (CAZy) database (73) implemented in dbCAN2 (105). The percentage of total annotated genes assigned to each gene family was calculated and is displayed in a multibar chart in iTol (99).

Additional metabolic annotations were assigned to the 67 *Bacteroidetes* sorted-MAGs using the GhostKoala server (82). Following annotation of protein coding genes, assigned knockouts (KOs) were used to estimate the completeness of selected pathways using KEGG-Decoder and a heat map was generated using “static” visualization mode to depict the completeness of each pathway (83).

### Data availability.

The bacterial and archaeal MAG data sets generated and analyzed in this study were deposited at NCBI GenBank under BioProject accession number PRJNA608716 and at https://bitbucket.org/lvalteio/forest_soil_mags_and_sortedmags/src, together with sequence alignments and phylogenetic trees generated in this study. Metagenomes and their corresponding metadata are available at IMG/M (https://img.jgi.doe.gov/m) under the taxon OIDs (identification numbers) indicated in Table S1.
