# Supplementary figures and images for: Complementary Metagenomic Approaches Improve Reconstruction of Microbial Diversity in a Forest Soil
Source: mSystems. 2020 Mar 10;5(2):e00768-19. doi: 10.1128/mSystems.00768-19 (PMC7065516; doi:10.1128/mSystems.00768-19)

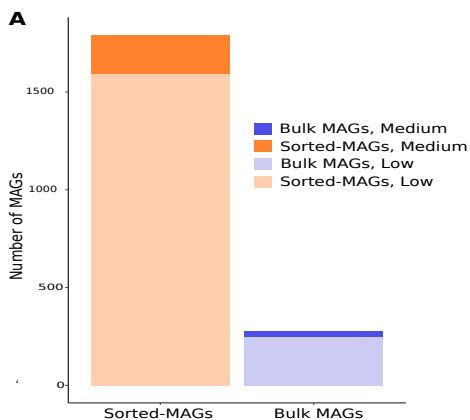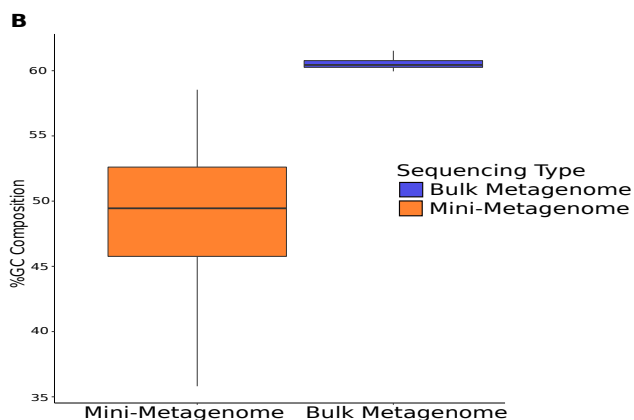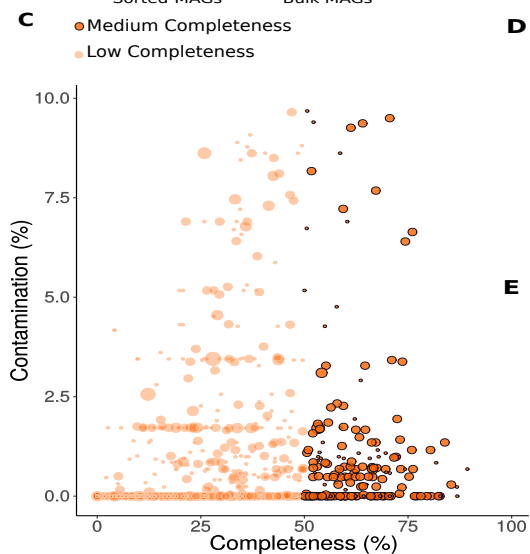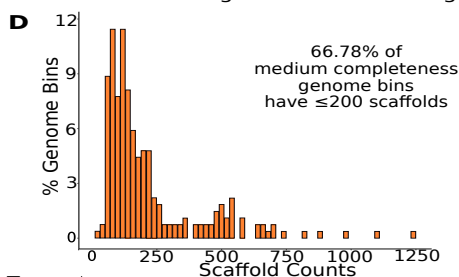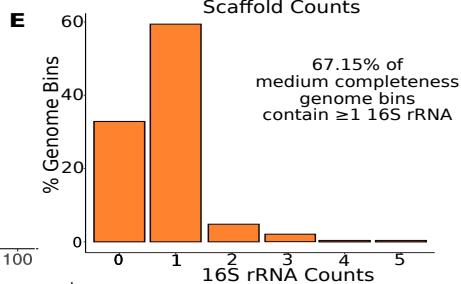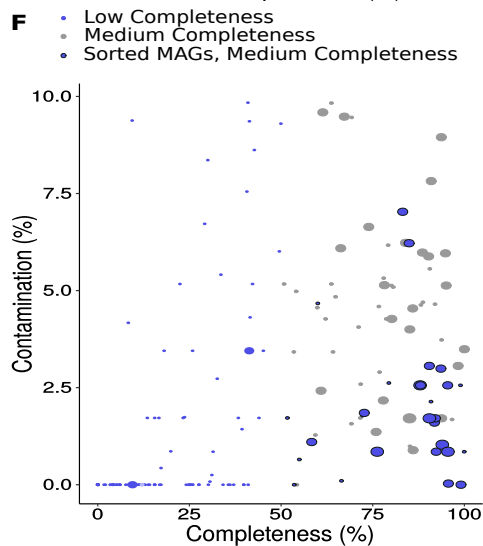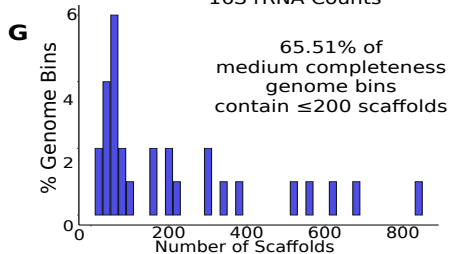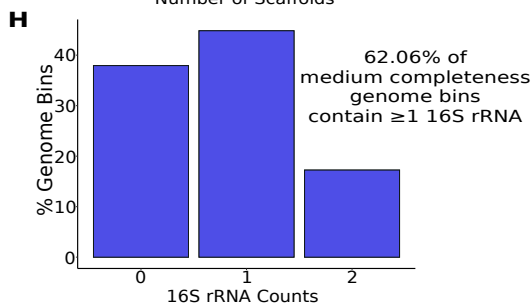

Supplement: FIG S1 [file mSystems.00768-19-sf001.pdf]

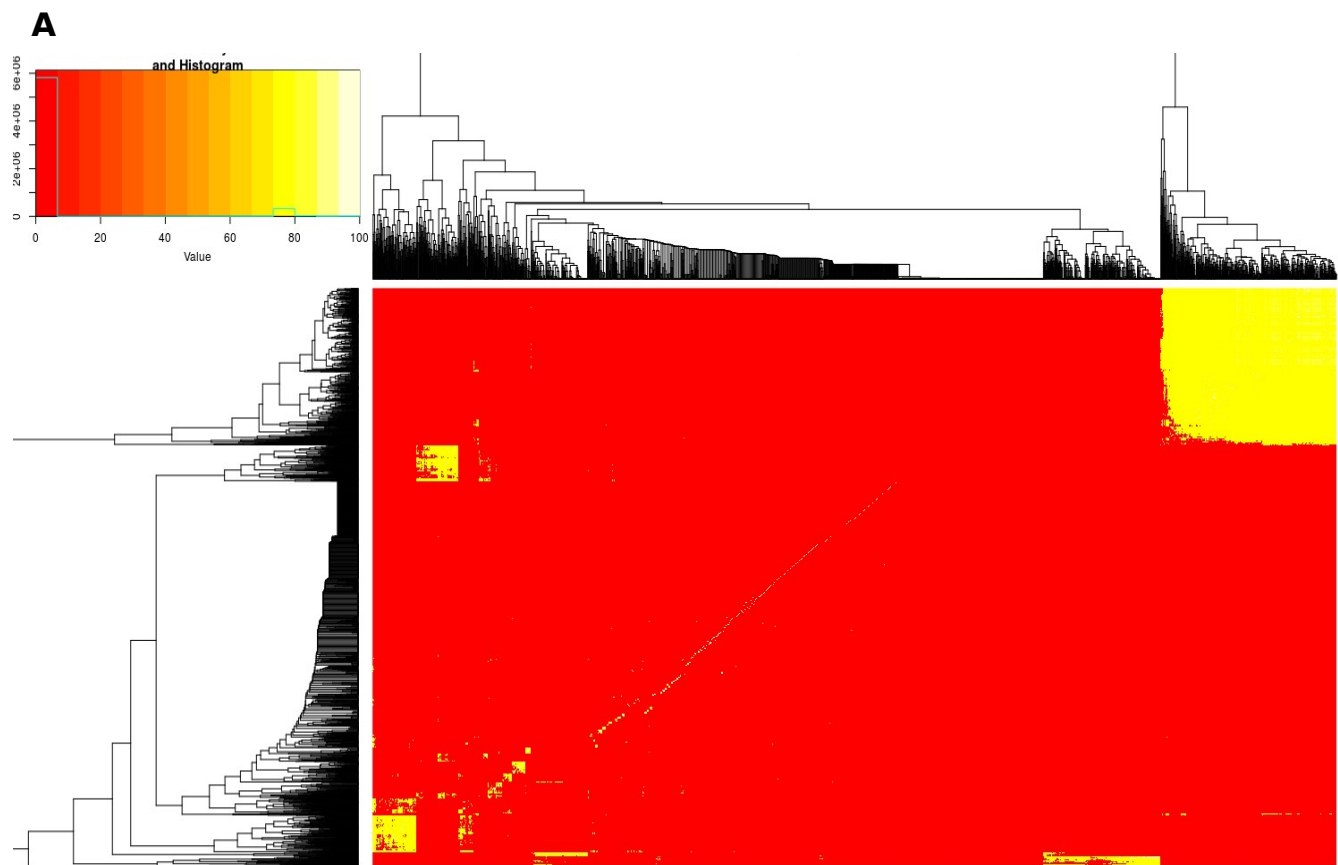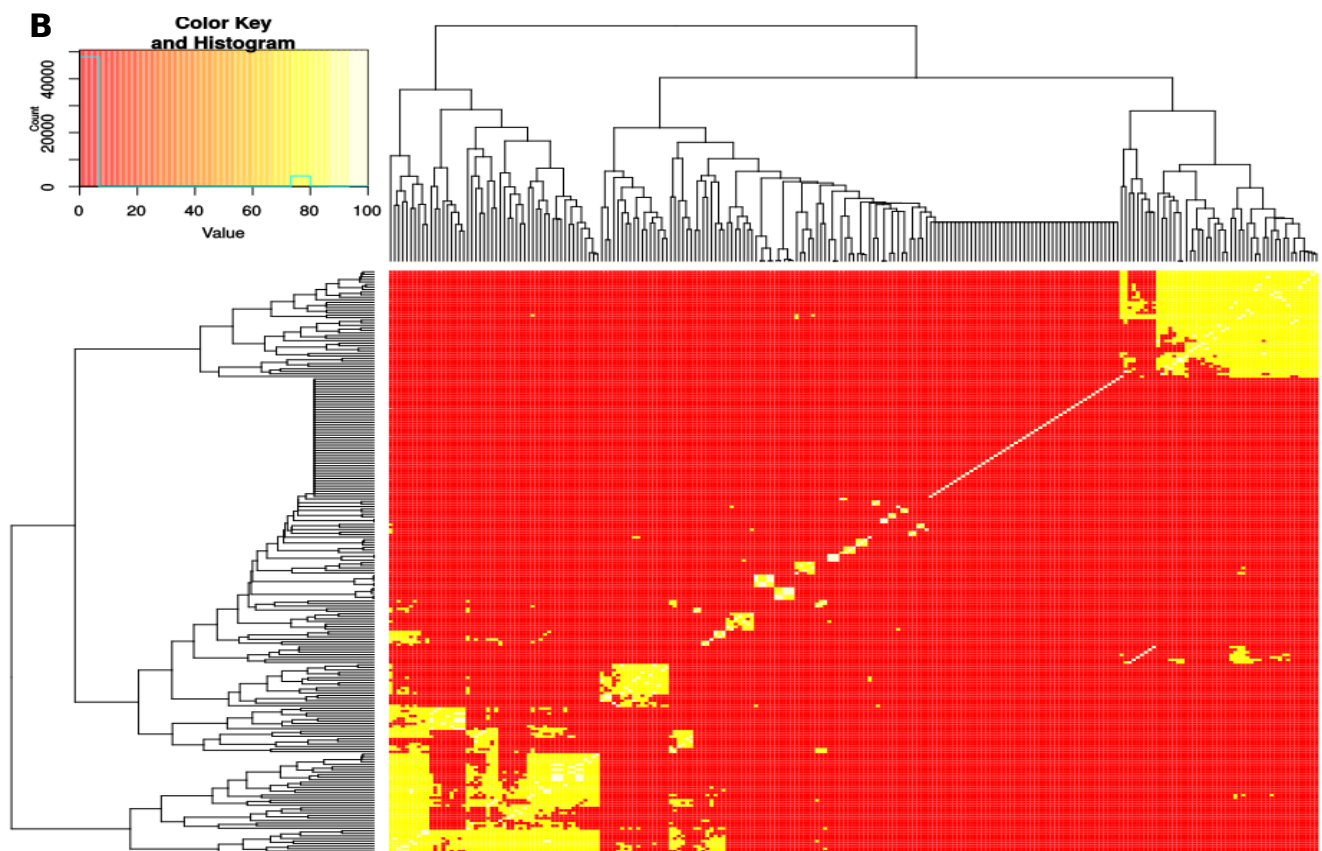

Supplement: FIG S2 [file mSystems.00768-19-sf002.pdf]

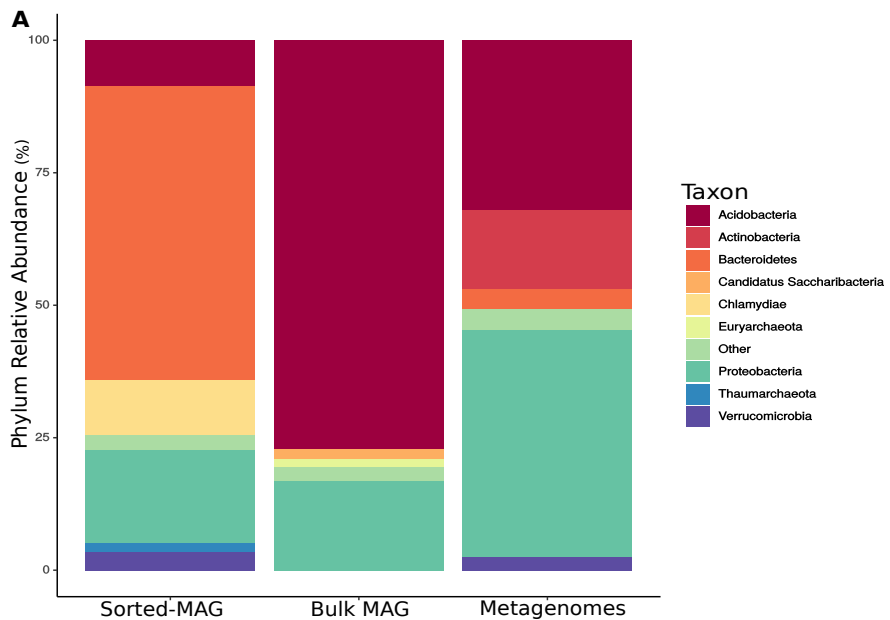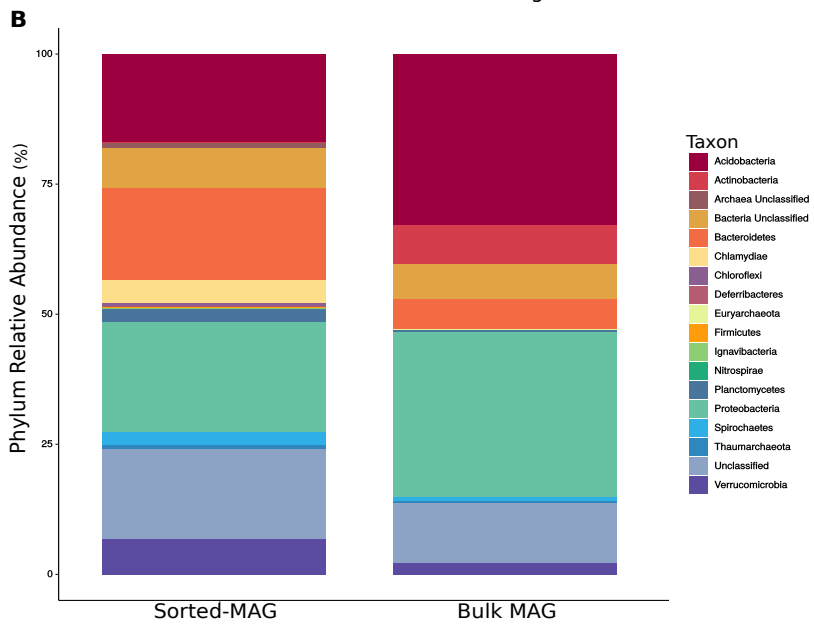

Supplement: FIG S3 [file mSystems.00768-19-sf003.pdf]

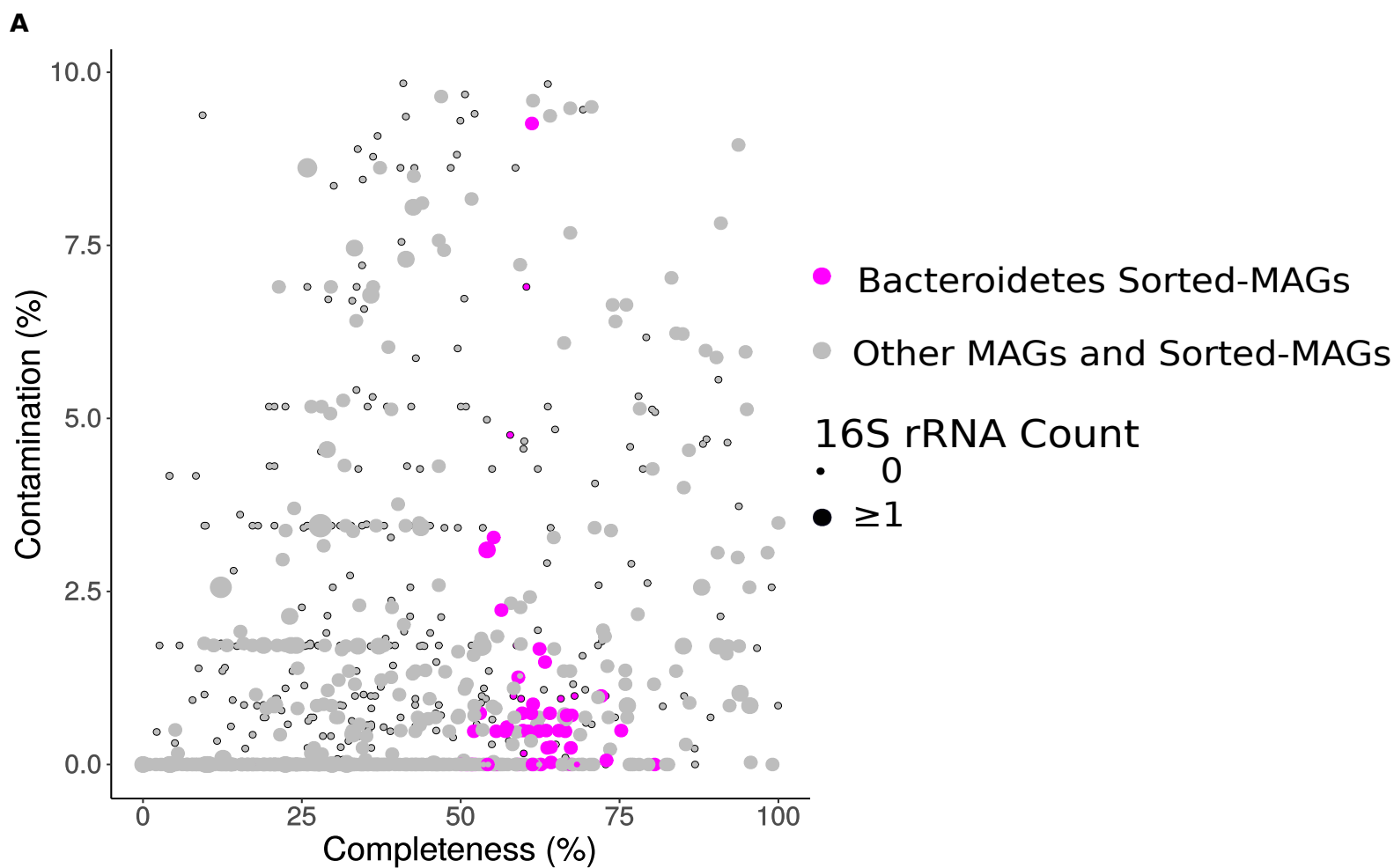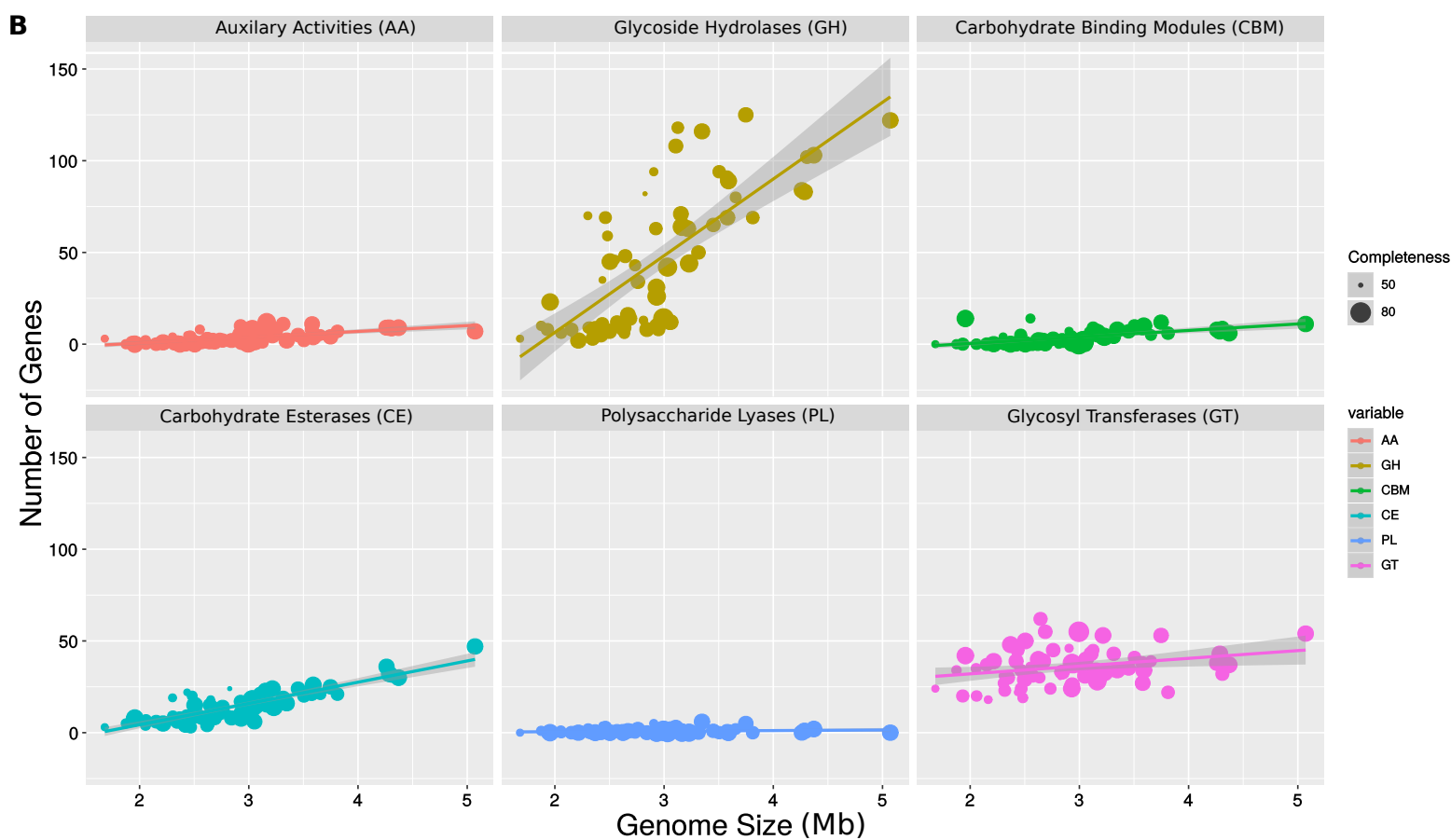

Supplement: FIG S4 [file mSystems.00768-19-sf004.pdf]

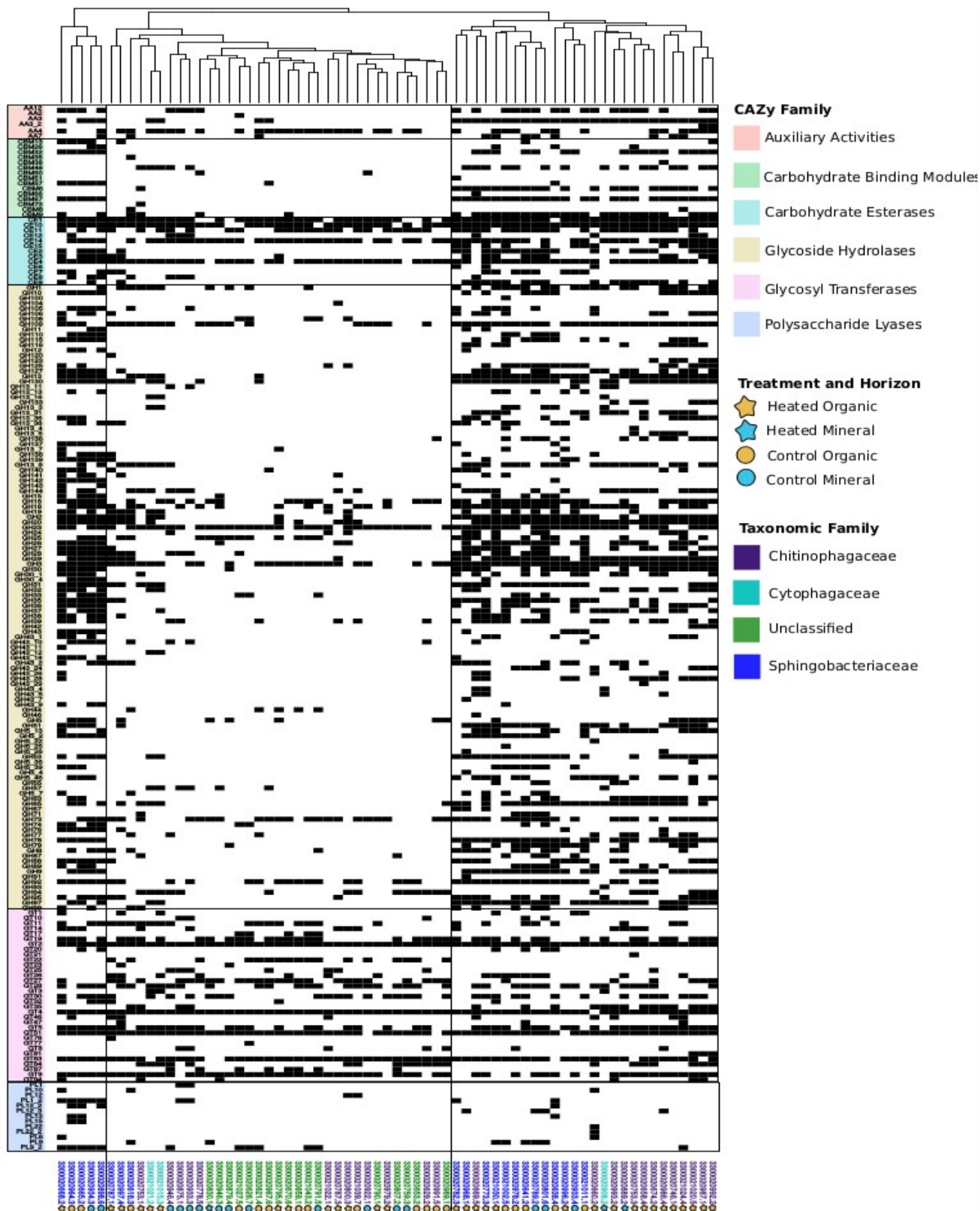

Supplement: FIG S5 [file mSystems.00768-19-sf005.pdf]

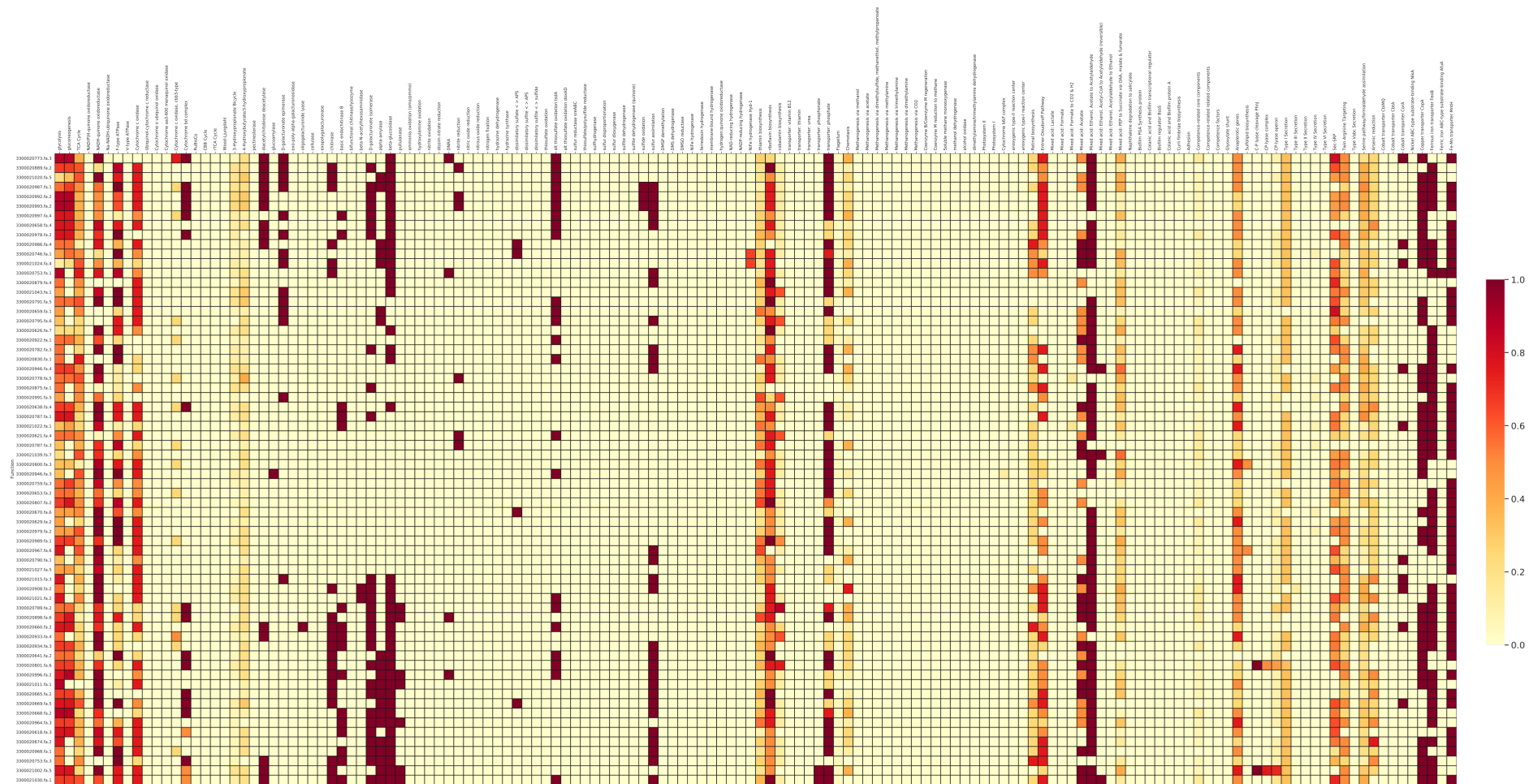

Supplement: FIG S6 [file mSystems.00768-19-sf006.pdf]
